# An *in vitro* comparison of intra-operative isohemagglutinin and human leukocyte antigen removal techniques in pediatric heart transplantation

**DOI:** 10.1051/ject/2023034

**Published:** 2023-12-15

**Authors:** Emily A. Hayes, Ashley B Walczak, Erin Goodhue Meyer, Kathleen Nicol, Matthew Deitemyer, Vicky Duffy, Michelle Moore Padilla, Robert J. Gajarski, Deipanjan Nandi

**Affiliations:** 1 The Heart Center, Nationwide Children’s Hospital Columbus OH 43205 USA; 2 Department of Hematology/Apheresis, Nationwide Children’s Hospital Columbus OH 43205 USA; 3 Department of Pathology, Nationwide Children’s Hospital Columbus OH 43205 USA; 4 Division of Cardiology, Department of Pediatrics, Emory University School of Medicine Atlanta GA 30322 USA

**Keywords:** Isohemagglutinin, Human Leukocyte Antigen, Plasmapheresis, Pediatric Heart Transplantation

## Abstract

*Background*: Highly sensitized pediatric patients awaiting heart transplantation experience longer wait times and thus higher waitlist mortality. Similarly, children less than 2 years of age have increased waitlist times and mortality when compared to their older peers. To improve the likelihood of successful transplantation in these patients, various strategies have been utilized, including peri-operative plasmapheresis. However, limited data exists comparing plasmapheresis techniques for antibody reduction. This study’s aim was to compare the *in vitro* magnitude of isohemagglutinin titers (IT) and human leukocyte antigen (HLA) antibody removal and the time required between membrane-based plasmapheresis (MP) and centrifuge-based plasmapheresis (CP) incorporated into the extracorporeal (EC) circuit. *Methods*: Two MP (Prismaflex) and two CP (Spectra Optia, Terumo BCT) circuits were incorporated into four separate EC circuits primed with high titer, highly sensitized type O donor whole blood. Assays were performed to determine baseline IT and anti-HLA antibodies and then at 30-minute increments until completion of the run (two plasma volume exchanges) at two hours. *Results*: There was a decrease in anti-A and anti-B IgM and IgG titers with both MP and CP. Mean anti-A and anti-B titer reduction was by 4.625 titers (93.7% change) and 4.375 titers (93.8% change) using MP and CP, respectively. At 2 h of apheresis, CP reduced 62.5% of all ITs to ≤ 1:4, while MP reduced 50% of ITs to ≤ 1:4. Additionally, reduction of anti-HLA class II antibody to mean fluorescence intensity (MFI) <3000 was achieved with both MP and CP. At 2 h of apheresis, CP reduced MFI by 2–3.5 fold and MP reduced MFI by 1.7–2.5 fold. Both demonstrated similar hemolytic and thrombotic profiles. *Conclusions*: In this *in vitro* plasmapheresis model of IT and anti-HLA antibody reduction, both MP and CP incorporated into the EC circuit can be used quickly and effectively to reduce circulating antibodies. While CP may have some greater efficiency, further study is necessary to verify this *in vivo*.

## Introduction

Heart transplantation remains the most effective treatment for patients with advanced heart failure refractory to medical management [1]. Over the past several decades, the outcomes following pediatric heart transplantation have continued to improve with a 1-year survival of over 90% [2]. However, the waitlist mortality in the United States remains high at 17%, ultimately limiting the use of this treatment in the pediatric patient population [3]. Furthermore, waitlist times are prolonged for smaller and highly sensitized children, leading to increased mortality in these patients [4].

In an effort to decrease waitlist mortality, strategies for the transplantation of highly sensitized patients and expanded use of ABO-I transplantation have progressed. One such strategy is the use of peri-operative plasmapheresis, including membrane-based plasmapheresis (MP) and centrifuge-based plasmapheresis (CP) [5]. Despite the common use of these techniques for both isohemagglutinin and HLA antibody removal, limited data exists to compare their effectiveness. This study aimed to compare the *in vitro* magnitude of isohemagglutinin titer (IT) removal and the time required between MP and CP incorporated into the extracorporeal (EC) circuit. While desensitization techniques incorporating MP and CP are utilized prior to transplant, the effects are variable in effect and duration and can be associated with serious complications [6, 7]. As transplant timing is difficult to predict, we focused on the feasibility of intra-operative plasmapheresis incorporated into the EC circuits.

## Methods

As highly sensitized, high titer donors were needed for this study, blood was obtained from the Red Cross from female donors with blood type O and at least one prior pregnancy. Twelve donor unites of whole blood were purchased and nine of the most sensitized units were pooled for a total volume of 3.5 L in a common reservoir. Heparin Sodium (Sagent Pharmaceuticals, Schaumburg, IL) was added to achieve a heparin concentration of 4.0 units/mL, which is standard dosing for cardiopulmonary bypass. Sodium Bicarbonate (Hospira, Inc., Lake Forest, IL) and Calcium Chloride (American Regent, Inc., Shirley, NY) were added to achieve physiologically normal blood gases. Once the common reservoir was homogenized (achieving a final hematocrit of 35–55%), aliquots of 450 mLs were utilized to prime each individual study circuit.

**Figure 1:**
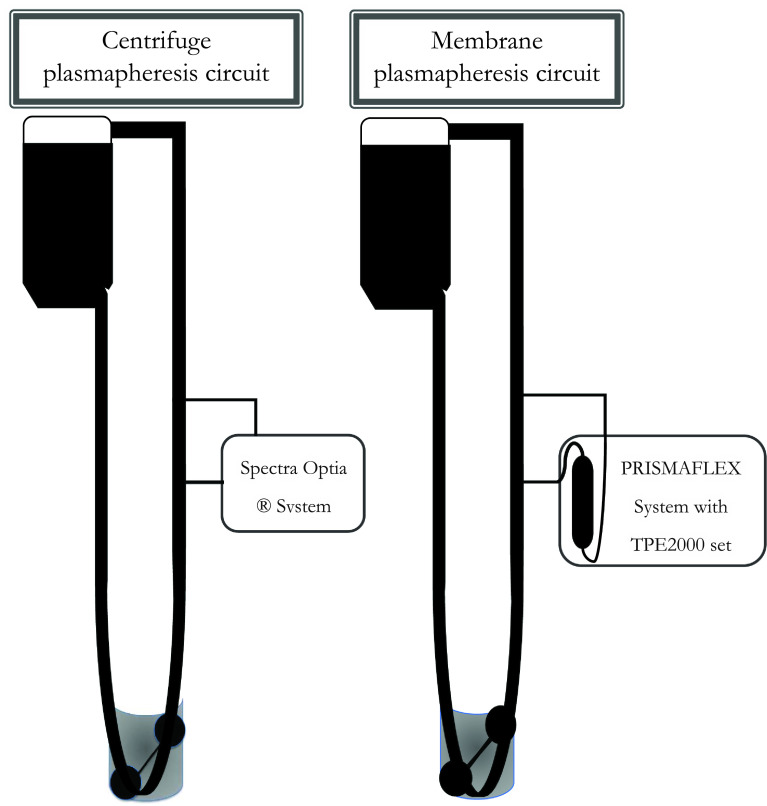
Centrifuge-based plasmapheresis (left) and membrane-based plasmapheresis (right) circuits incorporated into a cardiopulmonary bypass circuit.

Four identical extracorporeal (EC) circuits consisted of a Baby-Fx hard-shell reservoir (Terumo Cardiovascular, Ann Arbor, MI), a roller pump (Getinge, Goteborg, Sweden), and ¼ inch tubing (Terumo Cardiovascular, Ann Arbor, MI) creating a closed loop circuit. Two of the four study circuits incorporated a membrane-based plasmapheresis (Prismaflex, Baxter Healthcare Corporation, Deerfield, IL) circuit, and the other two study circuits incorporated a centrifuge-based plasmapheresis (Spectra Optia Apheresis System, Terumo Blood and Cell Technologies, Inc, Lakewood, CO) circuit, as demonstrated in Figure 1. The Prismaflex machine utilized a TPE2000 (Baxter) filter for this study.

Blood flow through each of the EC circuits was maintained at 500 mL/min to simulate a 2.2 L/min/m^2^ cardiac index for a 4-kilogram patient. The MP circuit flow rates were maintained between 100–400 mL/min and the CP circuit flow rates were maintained between 5–142 mL/min, according to the manufacturer’s instructions for use. Fresh Frozen Plasma (FFP) from AB+ donors wase used for the plasma exchange volume. The study was set to complete once 1.5–2× plasma exchange occurred or 2 h, whichever came first. Two hours was set as the average transplant time to simulate a true clinical scenario.

Baseline blood samples were analyzed for isohemagglutinin titers (IT), Panel Reactive Antibody (PRA) panel, complete blood count (CBC), and plasma hemoglobin. IT and a PRA panel were performed at 30-minute increments throughout the study. After the study, samples were again sent for final IT, PRA, CBC, and plasma hemoglobin. All samples were stored at four degrees Celsius until completion of the study, at which time they were delivered to the clinical laboratory for analysis.

## Results

There was a decrease in anti-A and anti-B IgM and IgG titers as well as a decrease in anti-HLA antibodies with both membrane-based and centrifuge-based plasmapheresis. Both demonstrated similar hemolytic and thrombotic profiles.

### Centrifuge-based plasmapheresis

**Figure 2:**
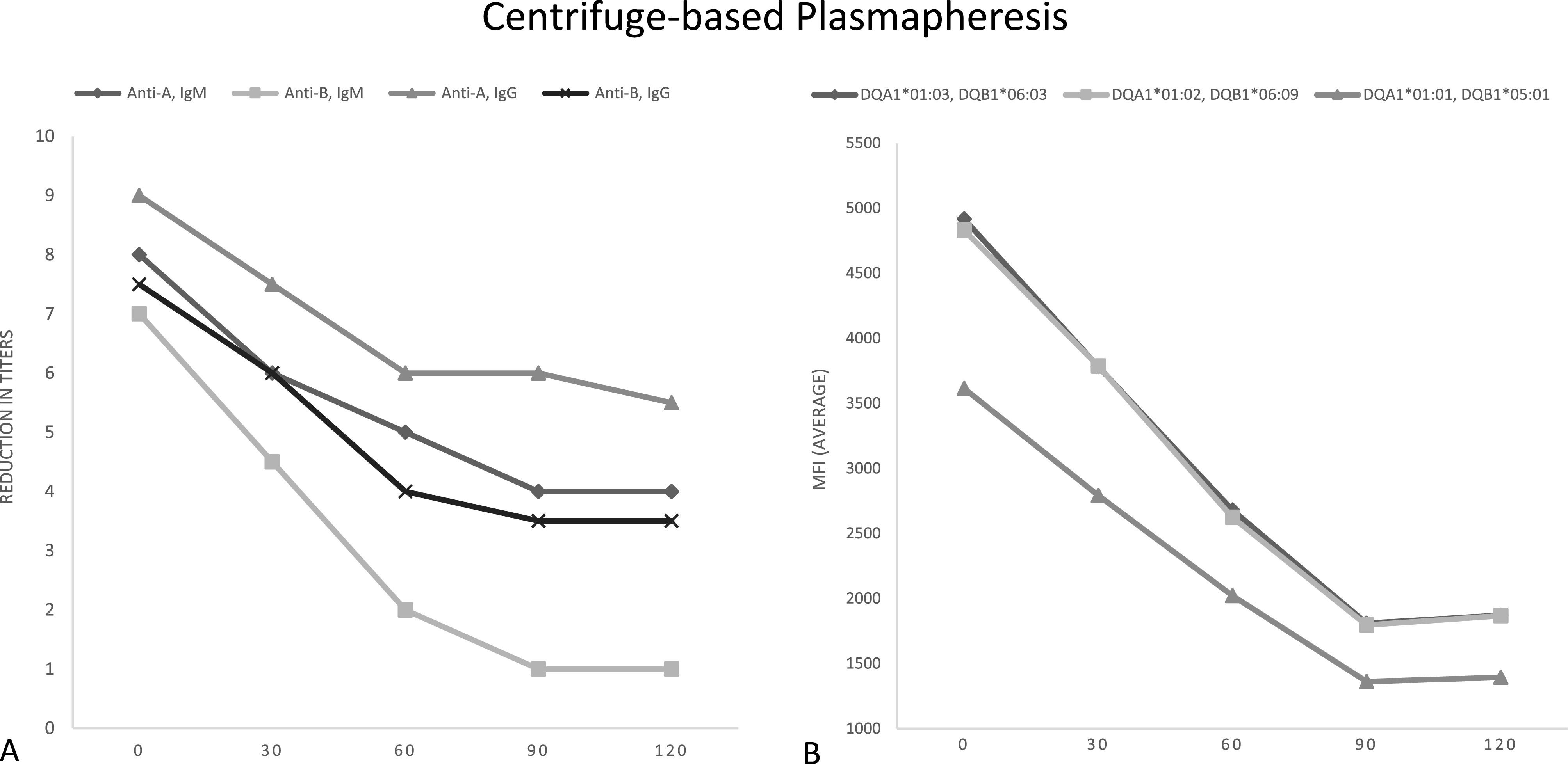
Centrifuged-based plasmapheresis results. A – Reduction in titers of anti-A and anti-B IgG and IgM. B – Reduction in HLA antibodies by average mean fluorescence intensity (MFI) at the DQ locus.

The mean baseline IT was 1:256 for anti-A IgM and 1:512 for anti-A IgG. Anti-B titers were lower for both IgM and IgG starting at 1:128 and 1:192, respectively. All four antibodies showed a decrease in titers with the first two passes and continued decrease or stable titers with the following two passes as demonstrated in Figure 2A. After the fourth pass, anti-A titers decreased to a mean of 1:20 for IgM and 1:48 for IgG, representing a reduction of 50% and 39%, respectively. Anti-B titers decreased to a mean of 1:2 for IgM and 1:12 for IgG, representing a reduction of 86% and 53%, respectively. The overall mean anti-A and anti-B titer reduction was 4.375 titers (93.8% change) with 62.5% of titers ≤ 1:4 at the end of the run at 2 h.

There were also high baseline class II anti-HLA antibody levels with all samples demonstrating a mean fluorescence intensity (MFI) >3,000. The cumulative MFIs prior to the runs were 12,106 and 14,615. Both CP circuits demonstrated a rapid decrease in MFI with a reduction to <3000 by the second pass at the 60-minute mark as demonstrated by Figure 2B. After the fourth pass, the cumulative MFI was reduced to 6,133 and 4,133 corresponding to a 2.0–3.5-fold decrease at the end of the two-hour run.

### Membrane-based plasmapheresis

**Figure 3:**
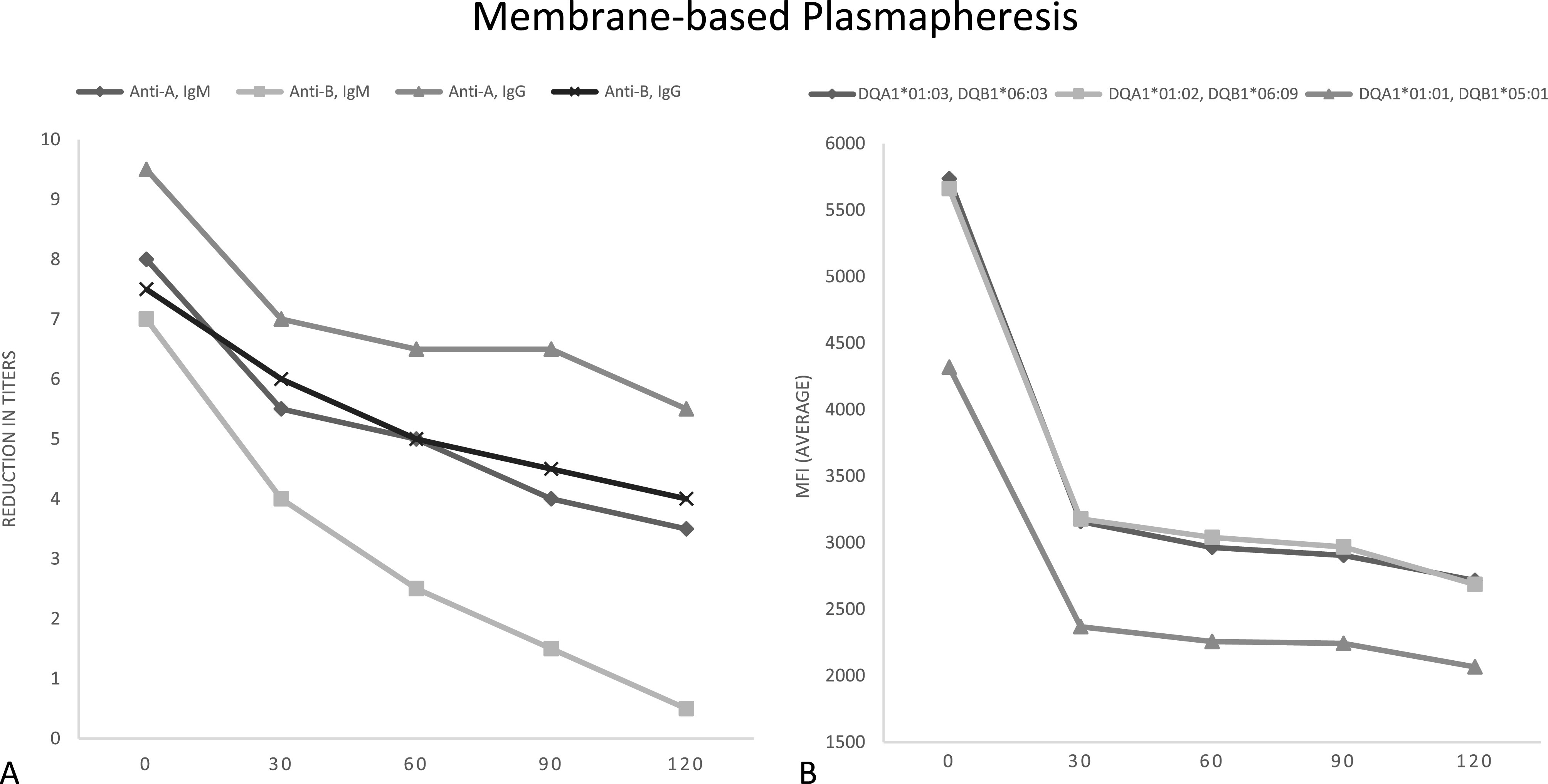
Membrane-based plasmapheresis. A – Reduction in titers of anti-A and anti-B IgG and IgM. B – Reduction in HLA antibodies by average mean fluorescence intensity (MFI) at the DQ locus.

The mean baseline IT was 1:256 for anti-A IgM and 1:768 for anti-A IgG. Baseline anti-B titers were again lower for both IgM and IgG starting at 1:128 and 1:192, respectively. Similar to CP, all four antibodies showed a decrease in titers with the first two passes. The following two passes demonstrated continued decrease or stable IT as demonstrated in Figure 3A. After the fourth pass, anti-A titers decreased to a mean of 1:18 for IgM and 1:72 for IgG, representing a reduction of 56% and 42%, respectively. Anti-B titers decreased to a mean of 1:1.5 for IgM and 1:20 for IgG, representing a reduction of 93% and 47%, respectively. The overall mean anti-A and anti-B titer reduction was 4.625 titers (93.7% change) with 50% of the titers ≤ 1:4 at the end of the run at two hours.

Furthermore, there were high baseline class II anti-HLA antibody levels with all samples demonstrating an MFI >3,000. The cumulative MFIs prior to the runs were 14,324 and 17,110. Both MP circuits demonstrated a rapid decrease in MFI with a reduction to <3,000 by the third pass at the 90-minute mark as demonstrated in Figure 3B. After the fourth pass, the cumulative MFI was reduced to 8,176 and 6,756, corresponding to a 1.7–2.5-fold decrease at the end of the two-hour run.

## Discussion

The presence of antibodies against HLA and ABO antigens and the historic association with rejection remains a barrier to successful transplantation for many patients [8]. There are various treatment strategies for antibody removal available to mitigate these risks at the time of transplant, including plasma exchange transfusion, plasmapheresis, and immunoabsorption. There is variable use of these strategies among transplant centers with no clear evidence that one strategy is associated with improved outcomes post-transplant.

Plasma exchange transfusion performed in the operating room prior to transplant remains the simplest and most cost-effective method to remove circulating antibodies. Prior to initiation of cardiopulmonary bypass, approximately 1.5–3 times the patient’s total plasma volume is exchanged for type AB donor plasma [9, 10]. However, there are several disadvantages to this strategy. Plasma exchange is not selective and therefore removes protective antibodies and clotting factors, increasing the risk of post-operative bleeding and infection. Additionally, the volume of blood products required limits use in larger patients and infers an increasing risk of transfusion-related mortality [11].

Alternatively, Robertson *et al.* recently published data utilizing immunoabsorption columns incorporated into the cardiopulmonary bypass circuit in ABO-I heart transplantation. With this method, a plasma separator and immunoadsorption column are incorporated into the EC circuit. After initiation of cardiopulmonary bypass, plasma is removed by the separator and filtered through the column with depleted plasma returned to the circulating volume of the EC circuit [12]. However, immunoabsorption columns are not readily available in many institutions in the United States. Furthermore, as this is a new technology, there is limited data on long-term outcomes.

Alternatively, plasmapheresis is commonly used in the peri-transplant period. MP utilizes a filter with pores that are large enough to remove the plasma and the desired macromolecules, whereas CP relies on the rotational forces of centrifugation to separate blood components based on their density [13]. Plasmapheresis circuits can also be incorporated into the EC circuit to remove a portion of the patient plasma that is then replaced with albumin or donor-fresh frozen plasma. We found that both membrane-based and centrifuge-based plasmapheresis incorporated into the EC circuit quickly and effectively reduced circulating IT and HLA antibodies. Both membrane-based and centrifuge-based plasmapheresis reduced anti-A and anti-B titers with similar efficacy with a reduction of 93.7% vs. 93.8%, respectively. Centrifuge-based plasmapheresis may have some greater efficiency in the removal of HLA antibodies with a 2.0–3.5-fold decrease compared to a 1.7–2.5-fold decrease for membrane-based plasmapheresis. Both membrane and centrifuge-based plasmapheresis utilized at the time of transplantation have the potential to expand the donor pool for older or highly sensitized children, though further study is needed to verify similar results *in vivo.*

## Conclusion

In this *in vitro* plasmapheresis model of IT and anti-HLA antibody reduction, both membrane-based and centrifuge-based plasmapheresis incorporated into the EC circuit can be used quickly and effectively to reduce circulating isohemagglutinin titers and anti-HLA antibodies. This technique has the potential to expand the donor pool for older or highly sensitized children. While centrifuge-based plasmapheresis may have some greater efficiency, particularly in HLA antibody removal, further study is necessary to verify this *in vivo*.

## Data Availability

The research data associated with this article are included within the article.
